# Association of Single Nucleotide Polymorphisms in the *ST3GAL4* Gene with VWF Antigen and Factor VIII Activity

**DOI:** 10.1371/journal.pone.0160757

**Published:** 2016-09-01

**Authors:** Jaewoo Song, Cheng Xue, John S. Preisser, Drake W. Cramer, Katie L. Houck, Guo Liu, Aaron R. Folsom, David Couper, Fuli Yu, Jing-fei Dong

**Affiliations:** 1 BloodWorks Northwest Research Institute, Seattle, WA, United States of America; 2 Department of Laboratory Medicine, Yonsei University College of Medicine, Seoul, Korea; 3 Human Genome Sequencing Center, Molecular and Human Genetics Department, Baylor College of Medicine, Houston, TX, 77030, United States of America; 4 Department of Biostatistics, University of North Carolina, Chapel Hill, NC, United States of America; 5 Department of Otolaryngology-Head and Neck Surgery, West China Hospital of Sichuan University, Chengdu, China; 6 Division of Epidemiology & Community Health, School of Public Health, University of Minnesota, Minneapolis, MN, United States of America; 7 Institute of Neurology, Tianjin Medical University General Hospital, Tianjin, 300052, China; 8 Division of Hematology, Department of Medicine, University of Washington School of Medicine, Seattle, WA, United States of America; Ohio State University Wexner Medical Center, UNITED STATES

## Abstract

VWF is extensively glycosylated with biantennary core fucosylated glycans. Most N-linked and O-linked glycans on VWF are sialylated. FVIII is also glycosylated, with a glycan structure similar to that of VWF. ST3GAL sialyltransferases catalyze the transfer of sialic acids in the α2,3 linkage to termini of N- and O-glycans. This sialic acid modification is critical for VWF synthesis and activity. We analyzed genetic and phenotypic data from the Atherosclerosis Risk in Communities (ARIC) study for the association of single nucleotide polymorphisms (SNPs) in the *ST3GAL4* gene with plasma VWF levels and FVIII activity in 12,117 subjects. We also analyzed *ST3GAL4* SNPs found in 2,535 subjects of 26 ethnicities from the 1000 Genomes (1000G) project for ethnic diversity, SNP imputation, and *ST3GAL4* haplotypes. We identified 14 and 1,714 *ST3GAL4* variants in the ARIC GWAS and 1000G databases respectively, with 46% being ethnically diverse in their allele frequencies. Among the 14 *ST3GAL4* SNPs found in ARIC GWAS, the intronic rs2186717, rs7928391, and rs11220465 were associated with VWF levels and with FVIII activity after adjustment for age, BMI, hypertension, diabetes, ever-smoking status, and ABO. This study illustrates the power of next-generation sequencing in the discovery of new genetic variants and a significant ethnic diversity in the *ST3GAL4* gene. We discuss potential mechanisms through which these intronic SNPs regulate ST3GAL4 biosynthesis and the activity that affects VWF and FVIII.

## Introduction

Von Willebrand factor (VWF) in the subendothelium tethers platelets to the site of vascular injury to initiate hemostasis, and protects coagulation factor VIII (FVIII) from enzymatic degradation [1;2]. VWF also contributes to thrombosis at the site of a ruptured atherosclerotic plaque and to platelet aggregation induced by high fluid shear stress in the area of severe vascular stenosis [3]. VWF and FVIII are synthesized primarily in endothelial cells [4–8]. Baseline levels of VWF and FVIII vary considerably among individuals and are regulated by genetic and environmental factors, including carbohydrate structures on the two molecules.

VWF and FVIII are extensively glycosylated. Each VWF monomer contains 13 potential N-linked and 10 O-linked glycosylation sites, with 4 additional glycosylation sites in the propeptide [9]. Together, the carbohydrates account for ~20% of the molecular mass of a VWF monomer [10] The complex types of biantennary core fucosylated glycans represent ~60% of the glycans on VWF, as compared to 13% represented by ABO-related glycans [11–13] Most of the N-linked and O-linked glycans on VWF are sialylated [12;14;15]. Enzymatically desialylated VWF is more adhesive [16;17] has an altered rate of cleavage by the metalloprotease ADAMTS-13 [18], and is rapidly cleared from the circulation through an asialoglycoprotein receptor [19]. Hypo-sialylated VWF is detected in the plasma of patients with pre-capillary pulmonary hypertension and those exposed to sialidase following microbial infection [19;20] FVIII is also glycosylated, with complex-type biantennary core fucosylated oligosaccharides, of which 80 to >90% carry at least one sialic acid [21–23] However, the functional importance of FVIII sialylation remains poorly understood.

Golgi-resident sialyltransferases of the ST3GAL family are type II membrane enzymes that catalyze the transfer of sialic acids in the α2,3 linkage to termini of N- and O-glycan chains. Six genes encoding these sialyltransferases have been identified in the mammalian genome (*ST3GAL1-4*) [24] Inactivating the murine St3gal4 sialyltransferase gene results in bleeding associated with an autosomal-dominant reduction in plasma VWF levels, with a minimal impact on VWF multimeric structures [25] The reactivity of the β-Gal-binding lectin RCA-I to VWF is increased in plasma from St3gal4-null mice because of an increase in the exposure of sub-terminal β-linked Gal on glycan branches [25] Similar results have been reported in rabbits and humans [26] indicating an important role of ST3GAL4 sialyltransferase in the biogenesis and survival of VWF and potentially of FVIII. The human *ST3GAL4* gene is located in the q23.3-q24 of Chromosome 11 [27], a locus that has been associated with the development of coronary artery disease [28] The gene spans more than 65 kilobases (kb), with 14 exons ranging from 61 to 679 nucleotides [27] There are 9 alternately spliced transcripts in the coding region of the human *ST3GAL4* gene, with tissue-specific patterns of expression [29]. The *ST3GAL4* RNA is widely expressed in cells and tissues, including megakaryocytes and platelets, with the highest levels being found in the small intestine and colon [25]. Variations in the human *ST3GAL4* gene remain undefined, and their influence on VWF, FVIII, and other sialylated proteins is not known.

We analyzed genetic and phenotypic data from the Atherosclerosis Risk in Communities (ARIC) study for the association of single-nucleotide polymorphisms (SNPs) in the human *ST3GAL4* gene with the plasma level of VWF and with activity of FVIII in 12,117 subjects. We also analyzed *ST3GAL4* SNPs from 2,535 subjects of 26 ethnicities from the 1000 Genomes (1000G) database, which has been interrogated with next-generation sequencing (NGS) technology for ethnic diversity, SNP imputation and construction of *ST3GAL4* haplotypes.

## Materials and Methods

### Study population

**ARIC** (https://www2.cscc.unc.edu/aric/desc) is an ongoing prospective cohort study designed to assess subclinical atherosclerosis and clinical atherosclerotic events [30] Baseline samples were collected from 1987 to 1989, from 15,792 adults aged 45 to 64 who were selected using probability sampling from Forsyth County, North Carolina; Jackson, Mississippi; the northwestern suburbs of Minneapolis, Minnesota; and Washington County, Maryland.

**The 1000G** (http://www.1000genomes.org/) project is designed to identify genetic variants that have frequencies of at least 1% in multi-ethnic populations using NGS. We analyzed *ST3GAL4* variants from the April 2012 Integrated Variant Set release of the 1000G project (ftp://ftp.1000genomes.ebi.ac.uk/vol1/ftp/release/20110521/ALL.wgs.phase1_release_v3.20101123.snps_indels_sv.sites.vcf.gz). The data were collected from 2,535 non-diseased subjects of 26 ethnicities and from four continents. Exonic regions of the genomes were sequenced at a high coverage rate (average >20X), and the whole genome was shotgun-sequenced at a low coverage rate (2–6X). The false discovery rate was estimated at 1.6% for exonic SNPs, 1.8% for non-coding SNPs, and <5% for insert-deletions (indels) [31]

### VWF antigen and FVIII activity

VWF antigen was determined with a commercial ELISA kit from American Bioproducts (Parsippany, NJ) and reported as a percentage of the Universal Coagulation Reference Plasma (Thromboscreen, Pacific Hemostasis, Curtin Matheson Scientific, Inc, Wooddale, IL) [32] The VWF measurement was taken during the first visits of ARIC subjects when they were recruited, from 1986 to 1989, and was, therefore, not defined as the international units widely used today. FVIII activity was measured using a commercial kit (George King Biomedical Inc. Overland Park, KA), defined as the ability of a testing plasma sample to correct the clotting time of human FVIII-deficient plasma, and reported as a percentage of normal plasma [33] The reliability coefficient (one minus intra-individual variance, divided by total variance), obtained from repeated tests on the same individuals over several weeks, was 0.68 for VWF and 0.86 for FVIII [32;33] The data were adjusted for covariates that are known to influence VWF and FVIII [33–35] including age, race, gender, body mass index (BMI), hypertension, diabetes, ever-smoking status, and ABO genotype.

### *ST3Gal4* SNPs and imputation

We analyzed *ST3GAL4* SNPs available from the ARIC GWAS and 1000G databases. The 1000G data allowed us to drastically increase the number of *ST3GAL4* SNPs to construct haplotypes using Haploview (http://www.broadinstitute.org/scientific-community/science/programs/medical-and-population-genetics/haploview). The 1000G SNPs and their haplotypes were imputed in the ARIC database separately for AA and EA samples using the IMPUTE2 program (https://mathgen.stats.ox.ac.uk/impute/impute_v2.html). The 1000G project’s phased genotype data for Europeans (n = 379) and Africans (n = 246) (https://mathgen.stats.ox.ac.uk/impute/impute_v2.html#reference) were used as references.

### Data analysis

All data were analyzed using SAS Proc LIFETEST or SAS Proc PHREG. The association of *ST3GAL4* SNPs with VWF antigen, FVIII activity, and FVIII–VWF ratio were evaluated using one-way ANOVA to test for mean differences among three genotypes of each SNP and for each of the four groups defined by race and gender (EA male, EA female, AA male, and AA female). A multiple linear regression model was used to define a relationship between genotype and outcome before and after adjustments for the covariates. We also tested ABO blood type by SNP interaction in the linear model in order to determine whether the association between SNP and outcome differs significantly by blood type, as these conform to different carbohydrate structures on the H antigen. With a Bonferroni adjustment for the number of SNPs tested, a p < 0.0038 was considered to be statistically significant for the association of a given genotype with VWF antigen and FVIII activity. A p-value between 0.05 and 0.0038 was considered to be nominally significant.

For each SNP, we also tested, through an additive model, to determine whether the number of minor alleles was additive. We also determined the allelic dosing effect by calculating whether having two copies of the allele had twice the effect of one copy (additive), larger than would be predicted by twice the effect of one copy (“more than additive”) or smaller effect than predicted by twice the effect of one copy (“below additive”). Finally, we also used HaploReg (http://www.broadinstitute.org/mammals/haploreg/haploreg.php) to predict the effect of 13 non-coding variants in intron 1 of the *ST3GAL4* gene on the regulatory regions of the gene.

## Results

### Subjects included in the study

Of the 15,792 ARIC participants, 3,675 were excluded from analysis because they (1) were neither European American (EA) nor African American (AA) (n = 48), (2) did not give consent for genetic studies (n = 45), or (3) randomly lacked the following data: FVIII activity, VWF antigen, or both (n = 280); *ST3GAL4* SNPs (n = 2607), or ABO genotypes (n = 640). The final analysis included 12,117 subjects. Table 1 reports the observed means for VWF, FVIII and the FVIII–VWF ratio by race and sex group for these subjects. Consistent with previous reports [35;36] VWF levels and FVIII activities varied significantly among the four race-by-gender groups. AA subjects had significantly higher VWF levels and FVIII activity than EA subjects (p < 0.001 and p < 0.003, respectively). Females had slightly higher VWF levels and FVIII activity than males. The ABO blood groups for the race-by-sex groups are listed in S1 Table.

**Table 1:** Mean (SD) of VWF antigen and FVIII activity by race and gender.

	Overall (N = 12,117)	EA-Female (N = 4914)	EA-Male (N = 4320)	AA-Female (N = 1800)	AA-Male (N = 1082)	*p* value (ANOVA)
**Factor VIII (%)**	130.0 (38.5)	127.2 (34.3)	122.7 (33.6)	149.0 (48.2)	140.9 (43.5)	<0.0001
**VWF (%)**	117.0 (46.9)	110.9 (42.1)	113.5 (43.2)	134.2 (56.6)	130.1 (54.4)	<0.001
**FVIII-VWF ratio**	1.19 (0.31)	1.22 (0.32)	1.15 (0.30)	1.20 (0.34)	1.16 (0.31)	<0.001

EA: Americans of European descent and AA: Americans of African descent

### *ST3GAL4* SNPs and genotype imputation

A total of 14 SNPs in the *ST3GAL4* gene (position: 126,355,645–126,652,917 on Chromosome 11) were identified in the ARIC GWAS database (Table 2; codes for the genotypes are listed in S2 Table). Among these, the SNPs rs2186717 and rs7928391 were completely linked (S3 Table). All 14 SNPs were genotyped in EA subjects, but only 4 SNPs were genotyped in AA subjects, and the rest were imputed. Thirteen of these SNPs (92.9%) were intronic, located in the first intron, and rs2298475 was exonic in exon 5.

**Table 2:** Position* and Allele frequency of *ST3GAL4* SNPs in ARIC-GWAS.

SNP	Location	Allele	Allele frequency		
			Major allele (A)	Aa	Minor allele (a)
rs629882	Intron 1	T/C	9656 (79.8%)	2248 (18.6%)	192 (1.6%)
rs3862628	Intron 1	G/A	6007 (49.6%)	4835 (39.9%)	1269 (10.5%)
rs3862629	Intron 1	C/T	5078 (57.3%)	3279 (37.0%)	502 (5.7%)
rs4601794	Intron 1	A/G	7588 (85.7%)	1219 (13.8%)	52 (0.6%)
rs11220463	Intron 1	A/T	7133 (80.5%)	1624 (18.3%)	102 (1.2%)
rs11220465	Intron 1	G/A	6256 (70.6%)	2369 (26.7%)	234 (2.6%)
rs7118117	Intron 1	A/G	5669 (46.8%)	4954 (40.9%)	1491 (12.3%)
rs2186717	Intron 1	T/C	2314 (26.1%)	4443 (50.2%)	2102 (23.7%)
rs7928391	Intron 1	C/T	2314 (26.1%)	4443 (50.2%)	2102 (23.7%)
rs10790800	Intron 1	A/G	4825 (39.9%)	5415 (44.7%)	1865 (15.4%)
rs7395043	Intron 1	T/C	6434 (72.6%)	2212 (25.0%)	213 (2.4%)
rs12574844	Intron 1	G/A	7462 (84.2%)	1325 (15.0%)	72 (0.8%)
rs11220476	Intron 1	C/T	6434 (72.6%)	2212 (25.0%)	213 (2.4%)
rs2298475**	Exon 5	T/C	7476 (84.4%)	1313 (14.8%)	70 (0.8%)

*SNP positions were defined based on UCSC Genome Browser on Human Feb. 2009 (GRCh37/hg19) Assembly (http://genome.ucsc.edu)

**Synonymous SNP (Leu-Leu)

**Fig 1:**
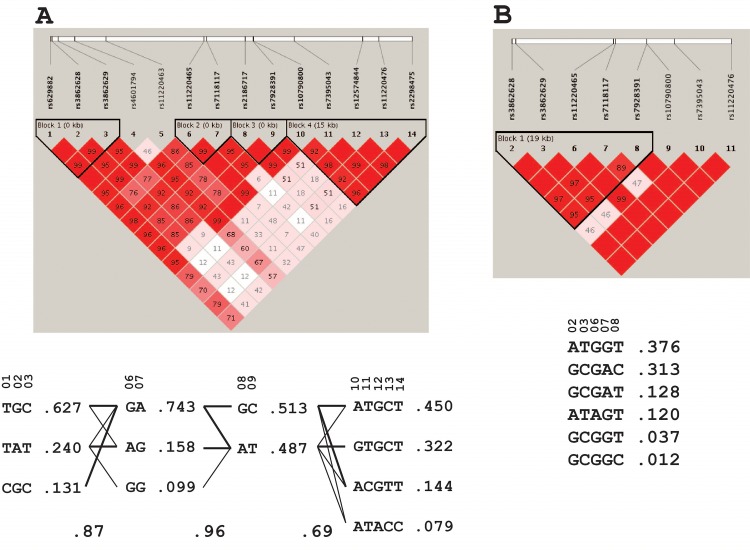
SNP density in the *ST3GAL4* gene. SNPs were plotted based the 1000 genomes data. The *ST3GAL4* gene has a very large first intron, where an overwhelming majority of SNPs are located.

To increase the number of *ST3GAL4* SNPs for association studies and to investigate their ethnic diversity, we also examined the 1000G database, which includes genotype data from 2,535 subjects of 26 ethnicities sequenced with NGS [37] Studying the ethnic diversity may be critical for causal effects of a given genetic variant as several studies [38;39], including our own [40], have shown that some of known VWF mutations associated with the bleeding disorder von Willebrand disease in EA subjects have minor allele frequencies of 10–20% in AA subjects, making them unlikely to cause the disease in African Americans. The findings suggest that the functional influence of a given variant can be enhanced or reduced by its association with specific haplotypes that are defined by ethnically diverse variations. We identified 1,714 variants in the *ST3GAL4* gene, including 986 novel variants whose identifiers were yet to be assigned by dbSNP. The locations of these SNPs in the *ST3GAL4* gene are shown in Fig 1. Eleven of the 14 *ST3Gal4* SNPs from the ARIC GWAS database were found in the 1000G database. Table 3 lists allele frequencies of these 11 SNPs among 10 representative ethnicities from America, Asia, Europe, and Africa, with 5 of them (46%) having significant ethnic diversity (>10-fold difference in allele frequencies among ethnicities, marked in grey).

**Table 3:** Ethnic allelic distribution (%) of *ST3GAL4* SNPs in 1000G*.

NP	FIN	TSI	CEU	YRI	LWK	ASW	CHB	JPT	MXL	CLM
rs629882	84.3	82.9	86.4	99.1	96.5	92.4	49.0	46.6	67.2	80.9
rs3862628	27.8	28.2	24.2	54.6	56.9	47.7	45.6	40.4	15.7	24.5
rs3862629	27.8	28.2	24.2	54.6	56.9	47.0	51.0	42.3	15.7	24.5
rs4601794	7.6	5.1	6.1	1.8	2.0	1.5	25.2	16.3	6.7	5.3
rs11220463	17.2	13.0	13.6	0.5	0.0	3.8	41.3	38.5	11.2	11.7
rs11220465	22.7	14.4	16.2	11.9	26.2	18.2	50.0	48.6	29.1	30.9
rs7118117	32.8	29.2	26.8	57.8	63.9	51.5	54.9	51.4	32.8	42.0
rs7928391	54.0	45.8	52.0	70.6	70.3	65.9	55.8	53.4	42.5	55.3
rs10790800	30.3	30.6	35.4	61.0	54.5	53.0	5.3	4.8	14.2	23.9
rs7395043	18.7	21.3	17.7	2.3	7.4	9.8	57.8	59.1	38.1	22.3
rs11220476	18.7	19.9	17.7	0.9	3.5	9.1	60.7	57.2	38.1	22.3

*This table lists reference alleles for **FIN**: Finnish from Finland; **TSI**: Toscani from Italy; **CEU**: Utah residents with Northern and Western European ancestry; **YRI**: Yoruba in Ibadan, Nigeria; **LWK**: Luhya in Webuye, Kenya; **ASW:** African Americans in Southwest US; **CHB**: Han Chinese from Beijing; **JPT**: Japanese from Tokyo; **MXL**: Mexican Ancestry in Los Angeles; and **CLM**: Colombian from Medellin Colombia.

**Fig 2:**
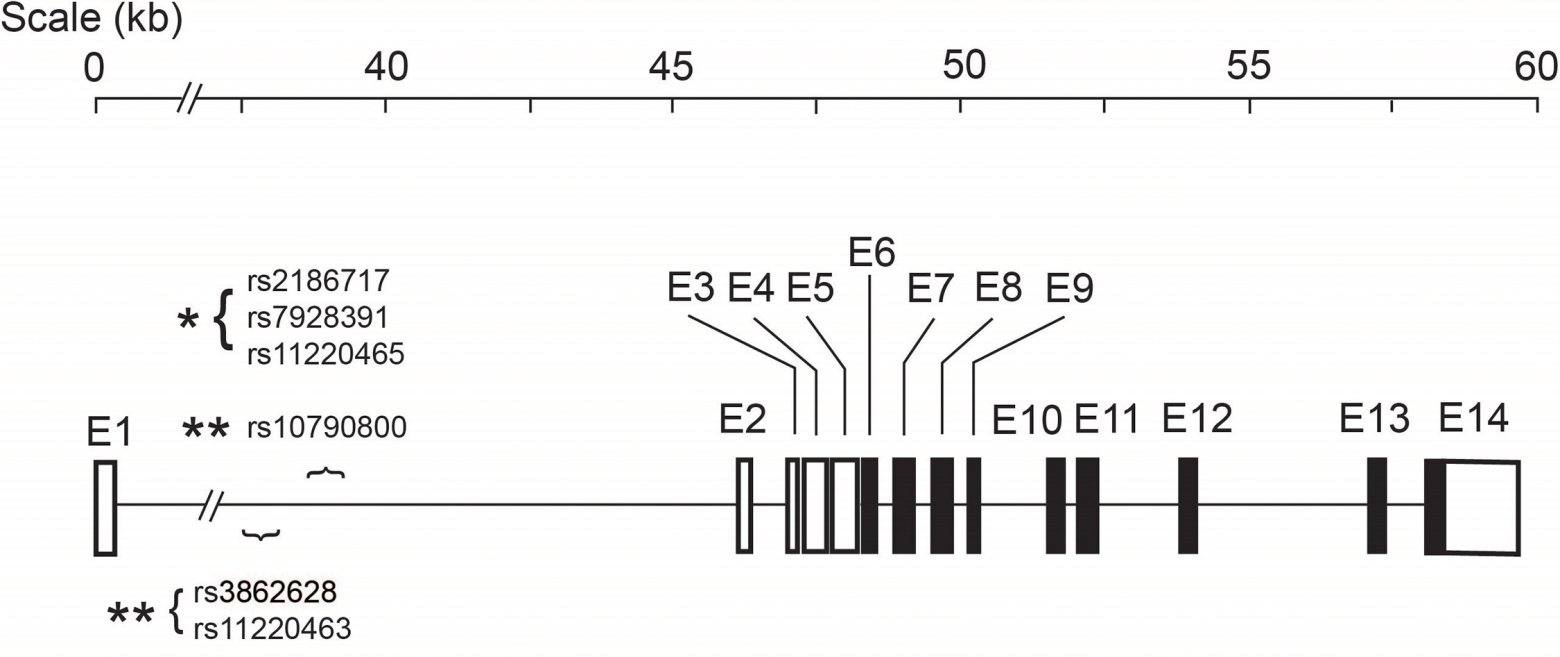
Linkage disequilibrium plot and haplotypes. Haplotypes and frequencies of *ST3GAL4* SNPs available in ARIC GWAS were analyzed separately for EA (**A**) and AA (**B**) samples.

Haplotypes of the *ST3GAL4* SNPs were constructed separately for EA and AA subjects (Fig 2). The SNP rs629882 was removed from subsequent analyses because it failed Hardy-Weinberg equilibrium testing (p-value = 5.19 × 10^−6^). We also used 1000G phased genotype data for European (EUR, n = 379) and African subjects (AFR, n = 246) for genotype imputations. The concordance rates defined by variations of alternative allele frequency between known genotypes of the *ST3GAL4* SNPs in ARIC GWAS and their imputed results from 1000G were 96.8% and 97.9% for AA (S4 Table) and EA (S5 Table) subjects, respectively, indicating a high imputation accuracy.

### Association of *ST3GAL4* SNPs with VWF antigen

Eleven *ST3GAL4* SNPs were significantly associated with the plasma level of VWF antigen before adjustments for age, BMI, hypertension, diabetes, ever-smoking status, and ABO (Table 4). The association remained for two completely linked SNPs (rs2186717 and rs7928391) after adjustment for these covariates. The SNPs rs11220465 and rs4601794 were associated with VWF levels with nominal significance. The stratified analyses further showed that only EA females had a statistically significant difference in mean VWF antigen levels across genotypes for the two linked SNPs. Multiple linear regression modeling for the ABO and SNP interaction suggested that the association between *ST3GAL4* SNPs and VWF was not modified by ABO blood groups.

**Table 4:** Association of *ST3GAL4* SNPs with VWF antigen (%) in ARIC*.

SNP		Genotype		P value^1^	P value^2^
	AA	Aa	aa		
rs7118117	106.9 (105.9,108.0)	108.8 (107.7,109.9)	114.3 (111.9,116.8)	< .0001	
	109.2 (108.2,110.2)	107.9 (106.9,109.0)	108.1 (106.1,110.0)		0.23
rs2186717**	101.4 (99.8,103.1)	105.4 (104.2,106.5)	105.4 (103.8,107.0)	0.0002	
	101.6 (100.2,103.0)	105.3 (104.3,106.3)	105.3 (103.9,106.7)		< .0001
rs7928391**	107.3 (105.8,108.8)	108.7 (107.6,109.8)	108.9 (107.5,110.4)	0.28	
	109.4 (108.0,110.8)	109.0 (108.1,110.0)	106.6 (105.4,107.8)		0.003
rs11220465	99.0 (94.6,103.6)	107.5 (106.0,108.9)	109.1 (108.2,110.1)	< .0001	
	101.7 (97.7,105.9)	108.2 (106.9,109.6)	108.7 (107.9,109.5)		0.006
rs3862629	106.8 (105.8,107.9)	109.1 (107.9,110.4)	113.7 (111.1,116.4)	< .0001	
	109.0(108.0,110.0)	108.0 (106.9,109.0)	107.4 (105.3,109.5)		0.26
rs3862628	113. 9(111.3,116.6)	109.1(108.0,110.3)	107.1(106.0,108.1)	< .0001	
	107.8 (105.7,109.9)	108.0 (107.0,109.1)	109.1 (108.2,110.1)		0.25
rs4601794	109.2 (108.4,110.0)	102.9 (100.7,105.0)	91.6 (81.8,102.5)	< .0001	
	108.6 (107.9,109.3)	106.9 (104.9,109.0)	98.7 (90.0,108.2)		0.04
rs7395043	109.0 (104.1,114.3)	105.5 (103.9,107.1)	109.2 (108.4,110.1)	0.0004	
	112.9 (107.9,118.1)	108.2 (106.7,109.7)	108.4 (107.6,109.1)		0.21
rs11220476	109.2 (108.4,110.1)	105.5 (103.9,107.1)	109.2 (104.3,114.5)	0.0004	
	108.3 (107.6,109.1)	108.2 (106.7,109.7)	113.2 (108.2,118.4)		0.17
rs11220463	109.3 (108.5,110.1)	103.9 (102.1,105.8)	102.3 (95.5,109.6)	< .0001	
	108.6 (107.8,109.3)	107.7 (105.9,109.5)	105. 6 (98. 8,112.8)		0.49
rs10790800	106.7 (105.6,107.8)	109.4 (108.2,110.5)	111.3 (109.2,113.4)	< .0001	
	108.8 (107.8,109.9)	108.8(107.8,109.8)	107.0 (105.3,108.7)		0.16

*Values presented are geometric means (95% confidence interval) and the shaded row for each SNP gives the adjusted values (N = 8,859 to 12,117 before and N = 8,790 to 12,006 after adjustment.

**RS2186717 and RS 7928391 are perfectly linked in EA, but data for RS2186717 is not available for AA subjects.

P values ^1^before and ^2^after adjustment for race, sex, age, BMI, hypertension, diabetes, ever smoking status and ABO

### Association of *ST3GAL4* SNPs with FVIII activity

We also identified 11 SNPs that were significantly associated with FVIII activity across genotypes before adjustment for the covariates (Table 5). The association with FVIII remained after adjustment for the covariates for rs2186717, rs7928391, and rs11220465, which were also associated with VWF after the adjustments. In addition, rs4601794 again showed nominally significant association with FVIII. ABO was not adjusted because it has a very weak influence on FVIII [41;42] None of the *ST3GAL4* SNPs had significant association with the FVIII–VWF ratio across genotypes (data not shown), but the p-value for rs2186717 and rs7928391 was 0.007 before adjustment for environmental covariates.

**Table 5:** Association of *ST3GAL4* SNPs with FVIII activity (%) in ARIC*.

SNP		Genotype		P value^1^	P value^2^
	AA	Aa	aa		
*rs7118117*	123.0 (122.1,123.9)	125.5 (124.5,126.5)	130.4 (128.4,132.4)	< .0001	
	125.5 (124.6,126.4)	124.6 (123.7,125.5)	123.5 (121.8,125.3)		0.13
*rs2186717*	118.8 (117.4,120.1)	120.8 (119.9,121.7)	121.6 (120.2,122.9)	0.01	
	118.6 (117.3,119.9)	120.9 (120.0,121.8)	121.4 (120.1,122.6)		0.006
*rs7928391*	113.3 (111.6,121.3)	119.6 (118.6,120.6)	125.7(121.8,128.8)	0.0007	
	128.1 (123.4,133.0)	125.6 (124.2,127.0)	124.6 (124.0,125.3)		0.002
*rs11220465*	115.3 (112.0,118.7)	124.2 (122.9,125.4)	125.5 (124.7,126.2)	< .0001	
	116.9 (113.3,120.7)	125.0 (123.8,126.2)	125.1 (124.4,125.8)		0.0002
*rs3862629*	123.0 (122.2,123.9)	125.8(124.8,126.8)	130.4 (128.1,132.6)	< .0001	
	125.3 (124.4,126.2)	124.6 (123.7,125.6)	123.6 (121.7,125.5)		0.26
*rs3862628*	130.4 (128.2,132.7)	125.7 (124.7,126.7)	123.1 (122.3,124.0)	< .0001	
	123.7 (121.9,125.6)	124.6 (123.7,125.6)	125.3 (124.5,126.2)		0.29
*rs4601794*	125.6 (124.9,126.3)	119.5(117.6,121.4)	108.9(101.1,117.3)	< .0001	
	125.0 (124.4,125.6)	123.9 (122.1,125.8)	114.3 (106.4,122.9)		0.03
*rs7395043*	122.7 (118.5,127.1)	122.1 (120.8,123.4)	125.7 (124.9,126.4)	< .0001	
	126.9 (122.5,131.5)	125.5 (124.1,126.8)	124.6 (124.0,125.3)		0.37
*rs11220476*	125.7 (124.9,126.4)	122.1 (120.8,123.4)	122.8 (118.5,127.1)	< .0001	
	124.6 (124.0,125.3)	125.5 (124.1,126.8)	127.0 (122.6,131.6)		0.37
*rs11220463*	125.8 (125.1,126.5)	120.0 (118.5,121.6)	115.2 (110.0,120.6)	< .0001	
	125.0 (124.3,125.6)	124.4 (122.8,126.1)	118.3 (112.3,124.6)		0.10
*rs10790800*	122.8 (121.9,123.8)	125.7 (124.8,126.7)	128.0(126.3,129.8)	< .0001	
	124.9 (124.0,125.9)	125.3 (124.4,126.2)	123.6 (122.7,125.1)		0.17

*Values presented are geometric means (95% confidence interval) and the shaded row for each SNP gives the adjusted values (N = 8,859 to 12,117 before and N = 8,790 to 12,006 after adjustment.

**RS2186717 and RS 7928391 are perfectly linked in EA, but data for RS2186717 is not available for AA subjects.

P values ^1^before and ^2^after adjustment for race, sex, age, BMI, hypertension, diabetes, ever smoking status. ABO was not adjusted.

We also measured allelic additive effects of these SNP for VWF antigen and FVIII activity by counting the number of minor alleles for each SNP. We identified several SNPs that were additive (Table 6). In addition, we also identified SNPs that were “more than additive” or “below additive”, which indicate that the additive effect was greater or less than what would be predicted by twice the effect of a single copy, respectively.

**Table 6:** Allelic additive effects of the *ST3GAL4* SNPs for VWF and FVIII*.

SNP	Additive for VWF	Additive for FVIII
RS7118117	Not additive	Additive
RS2186717	More than additive	Additive
RS7928391	Additive	Additive
RS11220465	Below additive	Below additive
RS629882	Not additive	Not additive
RS3862629	Not additive	Not additive
RS3862628	Not additive	Not additive
RS4601794	Additive	More than additive
RS7395043	Not additive	Not additive
RS12574844	Additive	Not additive
RS11220476	Not additive	Not additive
RS11220463	Not additive	Not additive
RS2298475	Additive	Additive
RS10790800	Not additive	Not additive

*After adjustments for confounding variables.

## Discussion

We analyzed the ARIC database for association of *ST3GAL4* SNPs with VWF antigen and FVIII activity. We also examined the 1000G database for genotype imputation and for ethnic diversity among *ST3GAL4* SNPs. The rationale for the study was: (1) the *ST3GAL4* gene encodes a sialyltransferase that adds sialic acids to termini of N- and O-glycan chains on a glycoprotein backbone, (2) VWF and FVIII contain biantennary core fucosylated glycans [11–13] and are modified by sialyltransferases [12;14;15] (3) removing sialic acids from VWF alters its biosynthesis and adhesive activity [43;44], and (4) inactivating the *ST3GAL4* gene results in a reduction in circulating VWF antigen in mice due to accelerated clearance [25] In this association study of two large adult samples, we made several novel observations.

First, 14 and 1,714 *ST3GAL4* SNPs were identified in the ARIC GWAS and 1000G databases respectively. The finding highlights the power of NGS for discovering new genetic variants. The density of variants in the ~65 kb *ST3GAL4* gene is consistent with that of the whole genome. However, the first intron, which contains approximately 75% of the nucleotides in the human *ST3GAL4* gene [27], contains all but one of the 14 SNPs found in the ARIC GWAS (Table 2) and an overwhelming majority of the SNPs found in the 1000G database (Fig 1). The large number of *ST3GAL4* variants found in 1000G permitted the construction of specific *ST3GAL4* haplotypes and allowed imputation to increase the detectable variants in the ARIC GWAS database. The study lays the foundations for studying more variations in the *ST3GAL4* gene and their associations with VWF biology and disease states when more data becomes available from ARIC exome and whole-genome sequencing.

Second, rs2186717, rs7928391 SNPs, and rs11220465 (the first two are perfectly linked) were associated with VWF levels and FVIII activity after adjustment for environmental covariates (Tables 4 & 5). The intronic SNPs rs2186717, rs7928391 SNPs, and rs11220465 are clustered in a region of less than 4000 bp in the first intron (<2% of the human *ST3GAL4* gene). The SNP rs4601794 shows a weak association, but it is close to the three clustered SNPs so that their effects, while minor individually, can be additive with or enhanced by other SNPs in specific haplotypes. In fact, this additive effect was found in 5 SNP for VWF antigen and 6 SNPs for FVIII activity (Table 7).

**Table 7:** Predicted motifs changed by non-coding variants in the *ST3GAL4* gene.

RS#	Location	Allele	Motif change
rs629882	Intron 1	C/T	Paired box 4, POU class 2 homeobox 2, SRY-related HMG-box
rs3862628	Intron 1	A/G	5 altered motifs
rs3862629	Intron 1	C/T	7 altered motifs
rs4601794	Intron 1	A/G	Nuclear receptor subfamily 3 group C member 1
rs11220463	Intron 1	A/T	B cell CLL/lymphoma, SINs transcription regulator family member 1
rs11220465	Intron 1	A/G	4 altered motifs
rs7118117	Intron 1	A/G	Zinc finger E-box binding homeobox 1
rs2186717	Intron 1	C/T	5 altered motifs
rs7928391	Intron 1	C/T	Runt related transcription factor 2
rs10790800	Intron 1	A/G	Sine oculis-related homeobox 5, trans-acting transcription factor 1
rs7395043	Intron 1	C/T	Transcription factor 12
rs12574844	Intron 1	A/G	Growth factor independent 1B transcriptional repressor, RE1 silencing transcription factor
rs11220476	Intron 1	C/T	B-cell CLL/lymphoma, estrogen receptor α-a, paired box 6

None of the SNPs is in a known intron-exon junction, so they unlikely affect RNA splicing. However, they may regulate ST3GAL4 biosynthesis and activity through other mechanisms. For example, rs2186717 is in a GT-rich sequence that is prone to homologous recombination [45] and is a preferred sequence for binding human DNA strand exchange protein, which is involved in the recombination process [46] Because of its location in the 5’-untranslated sequence [27] the first intron may also contain binding sites for transcription factors. A GT rich sequence has indeed been reported to be the substrate for the transcription factor Sp1 [47] which is reported to be important for transcribing the *ST3GAL1* gene [48] Similarly, the transcriptional regulation of the human *ST3GAL4* gene results in two mRNA species defined by transcription-factor binding to sites in the non-coding first exon and first intron [49]. Finally, using the in silico program HaploReg (http://www.broadinstitute.org/mammals/haploreg/haploreg.php), we were able to predict the effect of 13 non-coding variants in intron 1 of the *ST3GAL4* gene as regulatory SNPs (SNP effect on regulatory motifs, Table 6). These 13 variants can potentially modify at least one type of regulatory motif, suggesting that they are likely to have regulatory functions in haplotype blocks.

The genotype and phenotype associations identified in this study suggest that these SNPs influence the biogenesis and enzymatic activity of ST3GAL4 sialyltransferase, possibly causing differential sialylation on VWF and FVIII. Both VWF and FVIII are major sialylated proteins in the circulation, and their clearances are partly regulated by the asialoglycoprotein receptor-mediated endocytosis [19;25;50] In addition, *ST3GAL4* SNPs may affect the synthesis and survival of FVIII indirectly by regulating VWF, which forms a protective complex with FVIII in the circulation. As shown in Tables 4 & 5, the impacts of the individual correlative SNPs on VWF antigen and FVIII activity are small, but are probably additive because they are closely clustered. The small effect of the *ST3GAL4* SNPs may also be attributed to the compensatory activity of other homologous sialyltransferases and their alternative spliced forms.

In summary, we have identified novel SNPs in the *ST3GAL4* gene that are associated with VWF levels and FVIII activity. Although predominantly intronic, these SNPs may influence the synthesis and activity of ST3GAL4 sialyltransferase through different pathways. This association study lays the foundation for biological experiments to determine how these SNPs affect the expression and activity of the ST3GAL4 sialyltransferase. It will also be helpful to the study of variations in other genes in this family of sialyltransferases (*ST3GAL1-4*) [24] and their influence on the biogenesis and survival of VWF, FVIII, and other sialylated proteins.

**Key Point**

1. Human *ST3GAL4* gene is highly polymorphic and many of these polymorphic variants are ethnically diverse in an analysis of the 1000 genomes project database.
2. Six clustered SNPs in the first intron of the *ST3GAL4* gene are associated with plasma VWF level and FVIII activity.

## Supporting Information

S1 Table Frequencies (%) of ABO blood group by race and gender in ARIC. (TIF)

S2 Table Allele pairing codes for ARIC SNPs analyzed in the study. (TIF)

S3 Table Distribution of two completely linked SNPs. (TIF)

S4 Table Comparison of alternative allele frequencies (AAF) on *ST3GAL4* SNPs in AA samples before and after imputation using 1000G AFR reference panel. (TIF)

S5 Table Comparison of alternative allele frequencies (AAF) on 14 *ST3GAL4* SNPs in EA samples before and after imputation using 1000G EUR reference panel. (TIF)
