# Supplementary figures and images for: Association of Single Nucleotide Polymorphisms in the ST3GAL4 Gene with VWF Antigen and Factor VIII Activity
Source: PLoS One. 2016 Sep 1;11(9):e0160757. doi: 10.1371/journal.pone.0160757 (PMC5008807; doi:10.1371/journal.pone.0160757)

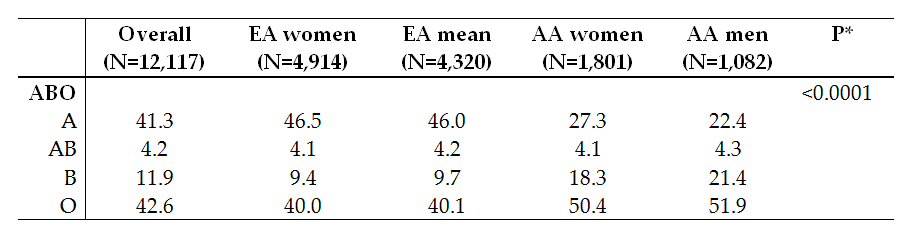

Supplement: S1 Table — (TIF) [file pone.0160757.s001.tif]

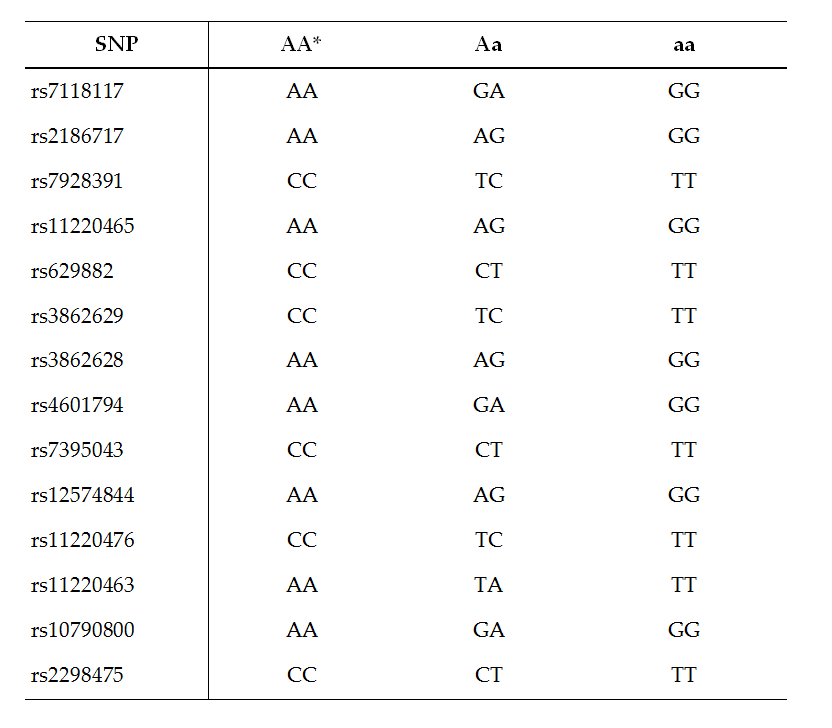

Supplement: S2 Table — (TIF) [file pone.0160757.s002.tif]

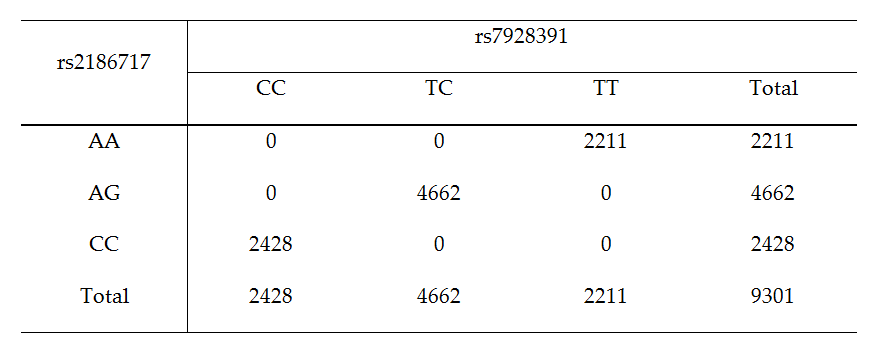

Supplement: S3 Table — (TIF) [file pone.0160757.s003.tif]

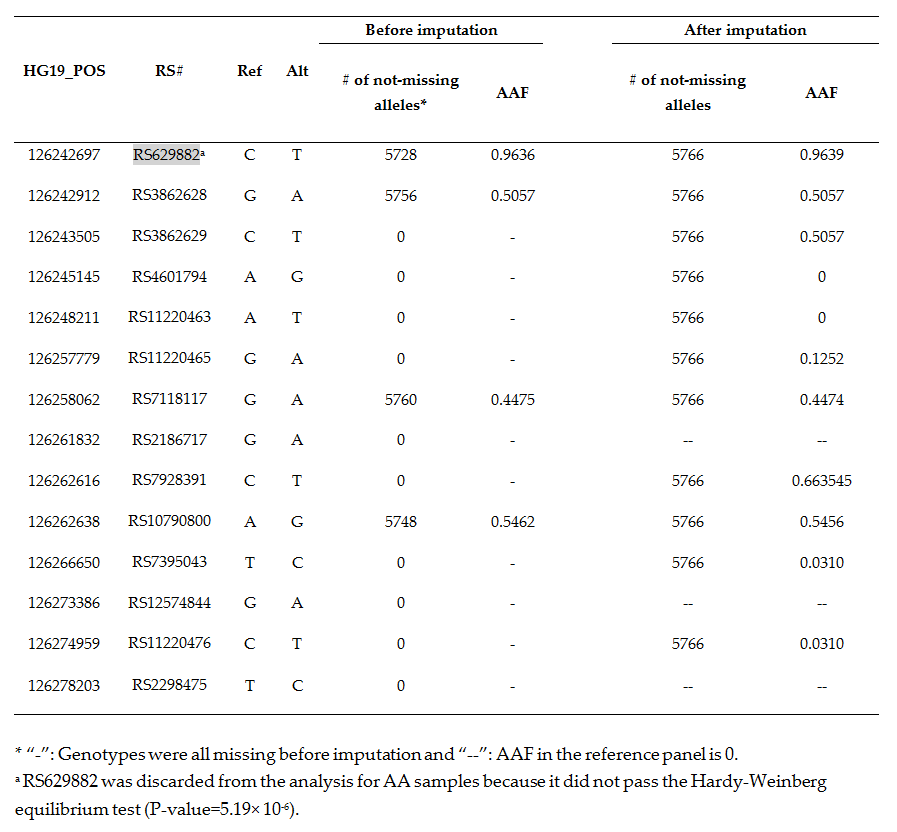

Supplement: S4 Table — (TIF) [file pone.0160757.s004.tif]

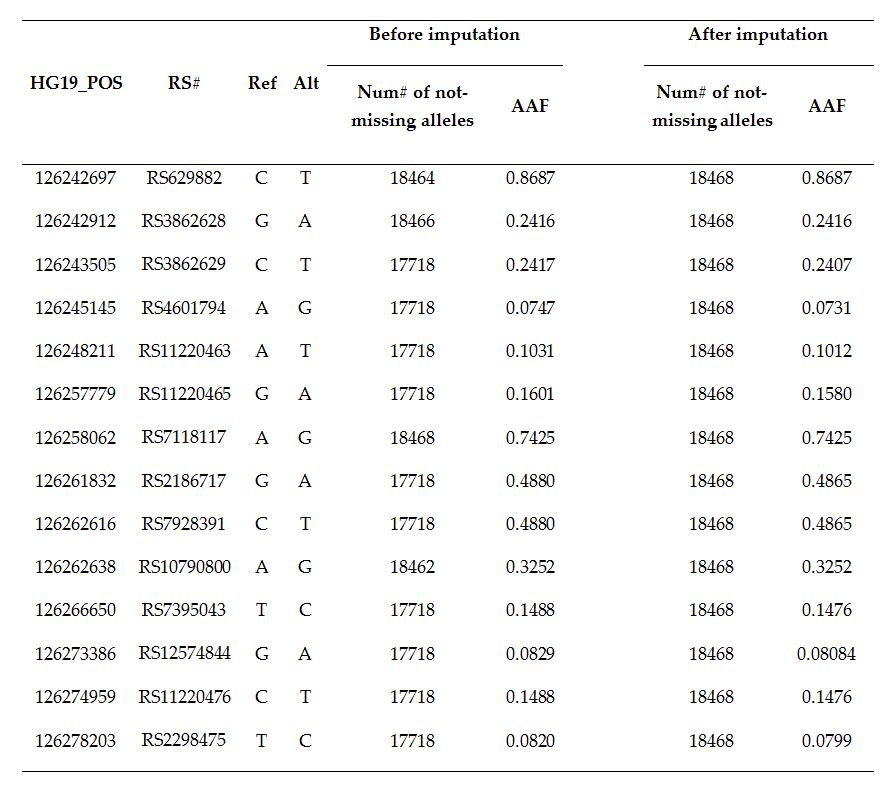

Supplement: S5 Table — (TIF) [file pone.0160757.s005.tif]
